# Immune microenvironment as a factor of breast cancer progression

**DOI:** 10.1186/s13000-015-0316-y

**Published:** 2015-06-26

**Authors:** Anatolii Romaniuk, Mykola Lуndіn

**Affiliations:** Department of Pathology, Sumy State University, m. Sumy, st. SKD 22-94, Sumy, Ukraine

**Keywords:** Breast cancer, Inflammatory infiltration, Receptors

## Abstract

**Background:**

The rate of progression of the disease depends on various factors and the tumor microenvironment takes not the last place among them. One part of researchers argues that the presence of tumor-infiltrating leukocytes serves as a favorable marker of the disease. There exists a completely different point of view on the matter.

The investigation of the effects of the inflammatory infiltration on the course of breast cancer process.

**Methods:**

We found a pronounced inflammatory infiltration in the tumor microenvironment in 24 cases. Nineteen cases of IDC without inflammatory infiltration were used as a control group. Immunohistochemical reaction showed expression of ERα, PR, HER2/neu, E-cadherin, Hsp90α, Bcl-2, CD3, CD79α, S100 and Myeloperoxidase receptors. Mathematical calculations were done using Microsoft Excel 2010 with 12.0.5 Attestat option.

**Results:**

We have determined five variants of immune microenvironment: interstitial, trabecular, nodular, diffuse and mixed. We have established a direct correlation between the expression of ER*α* and PR and indirect correlation between the receptors of steroid hormones and HER2/neo in both groups of breast cancer. HER2/neo positive tumors in 100% of cases were accompanied by the presence of heat shock proteins. There was a combination of Bcl-2 presence with the steroid receptors expression in 90 % of cases. There was found the indirect correlation between the presence of B lymphocytes and expression of steroid receptors.

**Conclusions:**

The presence of B lymphocytes in an inflammatory infiltrate leads to the disappearance of estrogen receptors and progesterone receptors. It provokes the accumulation of Hsp90 in a cell. It contributes to the stabilization of HER2/neu receptors and most proteins that promote tumor progression.

**Virtual slides:**

The virtual slides for this article can be found here: http://www.diagnosticpathology.diagnomx.eu/vs/1362330168161694

## Background

The rate of progression of breast cancer depends on various factors. The histological type of tumor, its reasons, the woman’s age, the hormonal condition, the tumor microenvironment, the status of the receptors and genetic material, etc. were described as the prognostic factors of breast cancer [1].

There are two different forms of estrogen receptor (ER), usually referred to as α and β, each encoded by a separate gene (ESR1 and ESR2, respectively). ESR1 is localized on the sixth chromosome, and ESR2 - on the fourteenth chromosome [2]. The connection of the hormone (17β-estradiol) with receptors causes dissociation of the complex with receptor protein hsp90, which impedes the transition of the receptor in the active conformational state. Further, as a heterodimer receptor it interacts with the estrogen response element (ERE), increasing the expression of the necessary genes [3]. The gene that encodes the progesterone receptor (PR) is located on the eleventh chromosome. The number of PR increases with the rising amount of estradiol and ER because they are synthesized after ER [4]. In contrast to these favorable prognostic indicators, the presence of human epidermal growth factor receptor 2 (HER2/neu) is a factor of tumor aggression. ERBB2 (coding gene HER2/neu), known as proto-oncogene, is located on a long arm of human seventeenth chromosome [5, 6].

The self-regulation tumorous process and participation of antitumor immunity in it are acute in many scientific studies. From this perspective the tumor microenvironment occupies a key position. One part of researchers argues that the presence of tumor-infiltrating leukocytes serves as a favorable marker of the disease [7]. The studies of the relationship between immune response and prevalence of breast cancer have shown that with the decrease of leukocyte infiltration around the tumor cells the frequency of regional lymph nodes lesion is increased [8]. On the one hand the immune microenvironment restricts prevalence of the tumor, on the other hand manifests its cytotoxic effect against the phenotypic-foreign breast cancer cells. There exists a completely different point of view on the matter [9]. It has been shown that the immune microenvironment determines the proliferative activity of the tumor, metastasis and cell migration. This process is carried out by the ability of cells to perceive signaling molecules such as pro-inflammatory cytokines, selectins, chemokines which are secreted by inflammatory cells as well as secrete them. But as it turned out the prognosis of breast cancer depends more on qualitative composition of the infiltration [10].

The questions about the influence of the inflammatory response on the expression of the receptors have been discussed in a great deal of studies repeatedly [11]. Nuclear factor (NF-kB) was described as one of the inhibiting factors for ER [12]. It is activated by a variety of stimuli, including cytokines (interleukin 1 - IL-1) and stress factors (such as reactive forms of oxygen). In the presence of IL-1, NF-kB becomes active. It is translocated into the nucleus for activation the transcription of the controlled genes. NF-κB can inhibit ER in different ways activating inhibitors and inhibiting activators of ESR1 and ESR2 transcription [13–15].

One important mechanism of cell survival is the activation of the transcription different anti-apoptotic proteins of the bcl-2 family by NF-kB signaling cascade [16, 17]. One of pro-survival proteins Bcl-2 family bcl-2 inhibits caspases due to the prevention of exiting cytochrome C from mitochondria and/or due to the binding factor APAF1 which activates apoptosis [18]. There is information that transcription of the gene Bcl-2 is activated by the signals received from the ER [19].

Heat shock protein 90 (hsp90, chaperone 90) is one of regulatory factors which controls activity of HER2/neu receptors [20]. It has been suggested that hsp90 modulates the effects of oncogenic HER2/neu, representing a potential mechanism of breast cancer resistance to HER2/neu directed drugs [21]. They are involved in maintaining the integrity of mitochondrial activating bcl-2, promoting the invasion step of metastasis and regulating the activity of factors Akt, NF-kB and JKN [22, 23]. Many investigations have shown the cross-resistance phenomenon in which some damaging agents (oxidase stress, heavy metal ions, inflammatory process) cause induction of hsp90 synthesis and adaptive response to other factors [24].

Analyzing the above it can be concluded that the inflammatory cells of the tumor microenvironment are directly involved in cell activity. The mechanism of combined effect immune cells on the expression of vitally important receptor of breast cancer cells remains unclear.

The **objectives** of this paper are as follows: the investigation of the effects of the inflammatory infiltration on the course of malignant process by determining qualitative composition of tumor microenvironment and studying the expression of the prognostic-important receptors in the breast cancer cells.

## Methods

We selected 43 samples of breast cancer tissue. The diagnosis of invasive ductal carcinoma (IDC) with varying degrees of malignancy (by Scarff-Bloom-Richardson) was found after the standard hematoxylin eosin staining of prepared slices. We found a pronounced inflammatory infiltration in the tumor microenvironment in 24 cases. Nineteen cases of IDC without inflammatory infiltration were used as a control group.

The material for the immunohistochemical reaction was fixed in 10 % neutral formalin for 24 h, after that paraffin blocks were made of it. Then sections with the thickness of 3–4 mm were made and they were subjected to the standard process of dehydration in xylol and alcohols of rising concentration. Immunohistochemical reaction consisted of 2 stages. During the first stage incubation with primary rabbit antibodies in different dilutions took place (Table 1). We used the following antibodies for determining the qualitative composition of infiltration: CD3 was used for the determination of T lymphocytes, CD79α – for B lymphocytes, the searching of the antigen presenting cells was performed using S100 antibody, myeloperoxidase (MPO) being detected in myeloid cells of both neutrophilic and eosinophilic types. We have performed immunohistochemical reaction with E-cadherin for the purpose of the differential diagnosis between invasive lobular carcinoma and high degree malignant IDC. The positive reaction took place only in IDC.

**Table 1:** Antibody for immunohistochemical reaction

Antibody	Host	Clone	Dilution	Cellular localization
ERα	Rabbit	SP1	1:200	Nuclear
PR	Rabbit	YR85	1:150	Nuclear
HER2/neu	Rabbit	SP3	1:100	Membrane
E-cadherin	Rabbit	67A4	1:100	Membrane
Hsp90α	Rabbit	Polyclonal	1:200	Cytoplasmic and nuclear
Bcl-2	Mouse	100/D5	1:100	Cytoplasmic
CD3	Rabbit	SP7	1:150	Membrane
CD79 α	Rabbit	SP18	1:200	Membrane
S100	Mouse	4C4.9	1:150	Cytoplasmic
Myeloperoxidase	Rabbit	Polyclonal	1:100	Cytoplasmic

The incubation with secondary antibodies (UltraVision ONE HRP Polymer) took place during the second stage. We visualized the cell structural components using diaminobenzidine, which painted them in a brown color.

We calculated the share of different forms of immune cells and their ratio analyzing the histological and immunohistochemical study. The study was conducted in 10 fields of view at 400× magnification.

Mathematical calculations were done using Microsoft Excel 2010 with 12.0.5 Attestat option. We have found such indicators as chi-squared Pearson’s test, Student’s T-criterion, Pearson’s correlation coefficient (with statistical significance *p* < 0.05).

## Results

At histological examination of cases with inflammatory infiltration we have found that immune cells are located in different variations around the tumor cells. Among the immune cells, we found that most cells had a rounded shape with a rounded nucleus and a small amount of cytoplasm of approximately 6mkm which were identified as lymphocytes. Considering histological features we were unable separate them on T or B lymphocyte groups. Among them we found the histiocytes which were characterized by the presence of various (round, elongated or irregular) forms, heterogeneous spotted basophilic cytoplasm (due to the presence of lysosomes) of approximately 13mkm. There were also found scattered segmented granulocytes various forms with contained intracellular granularity. We have determined five variants of immune microenvironment:

1. Interstitial - the leukocytes are located around the tumor as single scattered cells.
2. Trabecular – the immune cells form the layers or trabeculae.
3. Focal (nodular) – the leukocytes accumulate in focuses, sometimes with the formation of pseudo-follicles.
4. Diffuse - the immune cells occupy almost all the intercellular space giving the impression of metastasis in lymph nodes (Fig. 1).

**Fig. 1:**
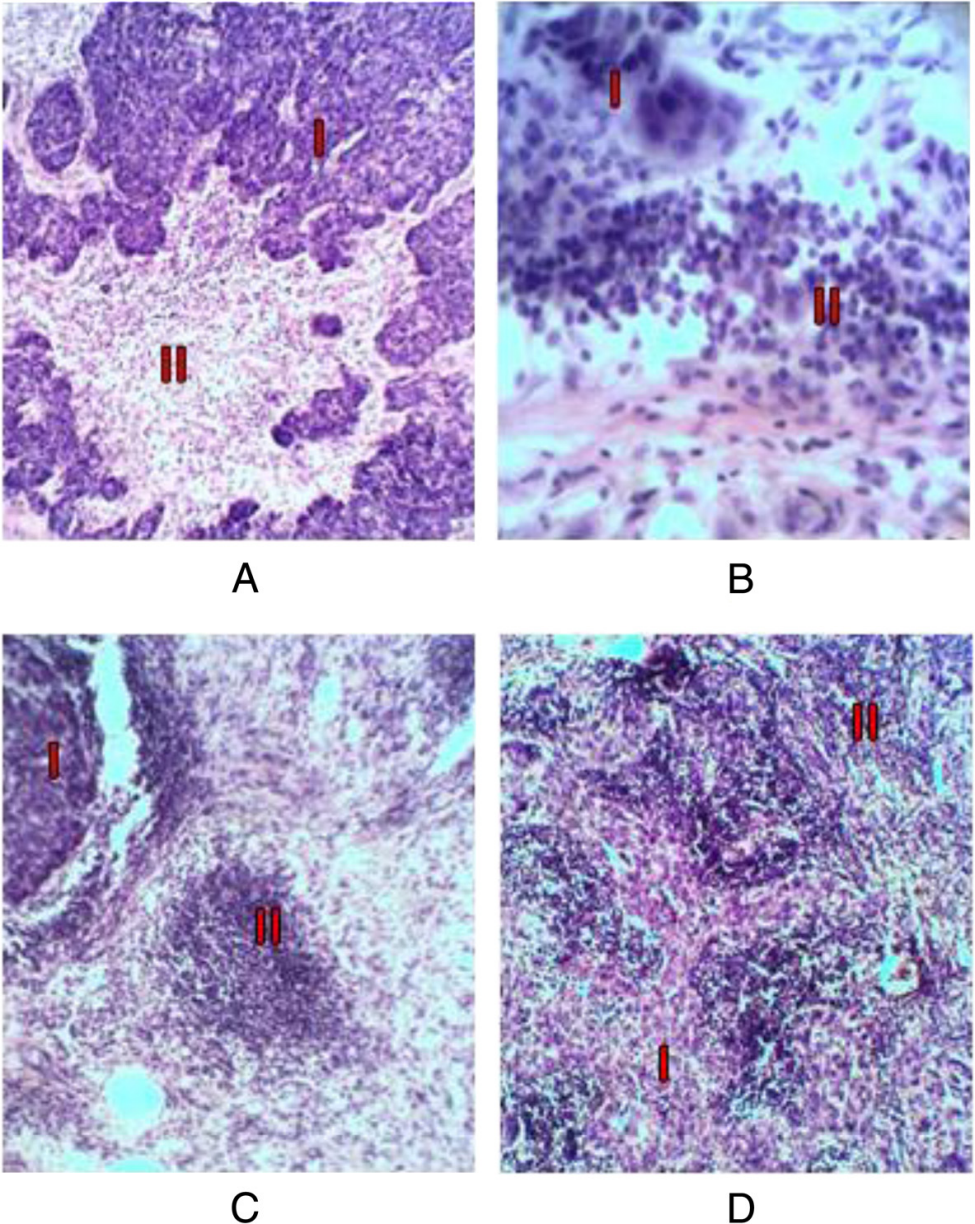
The variants of immune microenvironment: **a** – interstitial, **b** – trabecular, **c** – focal, **d** – diffuse. I – the tumor tissue; II - the immune cells. The hematoxylin eosin staining. Magnification × 100
5. Mixed – a combination of different forms of inflammatory infiltration.

In the stroma of the control group tissue the immune cells were also presented, because they are a part of the fibrous tissue of any organs, including the mammary gland. But they are located singly and very rare.

A clear relationship between the degree of malignancy of IDC and the type of inflammatory infiltrate has not been detected (*p* > 0.05). It was observed a tendency of infiltration increase in the case of the growth of polymorphism “cancerous” cells. This suggests a more pronounced reaction of macroorganism with the increase of cell’s anaplasia.

Immunohistochemical analysis has revealed different variants of ER*α*, PR and HER2/neu expression (Table 2).

**Table 2:** The molecular subtypes of breast cancer

		The tumors with inflammatory infiltration	The tumors without inflammatory infiltration
Luminal A	ER+	3	13
	PR+/−		
	HER2/neu−		
Luminal B	ER+	1	1
	PR+/−		
	HER2/neu+		
Triple negative	ER−	15	2
	PR−		
	HER2/neu+		
HER2 type	ER−	5	3
	PR−		
	HER2/neu−		

We have established a direct correlation between the expression of ER*α* and PR (*r* = +0.78, *p* < 0.05) and indirect correlation between the receptors of steroid hormones and HER2/neo (*r* = −0.73, *p* < 0.05) in both groups of breast cancer. The combination of the steroid receptors absence and presence of HER2/neo with the presence of inflammatory infiltration in the tumor microenvironment have been established in our study. In most cases, it did not depend on the type of inflammatory infiltration.

Studying the expression of bcl-2 in cancer cells the investigation has showed that 40 % of tumors were bcl-2 positive. There was a combination of their presence with the steroid receptors expression in 90 % of cases. It was found that hsp90α in tumor cells were characterized by cytoplasmic and nuclear localization. Nearly 80 % cases of breast cancer were positive for Hsp90α expression, indicating their participation in tumor development. The correlation was established between the level of hsp90α expression by tumor epitheliocytes and the leukocyte infiltration in stromal tissue (*p* < 0.05). HER2/neo positive tumors in 100 % of cases were accompanied by the presence of heat shock proteins.

While studying the qualitative composition of the inflammatory infiltration it has been found that the immune cells were white blood cells of different nature. The lymphocytes dominated among them. We have established that the lymphocytes of T (CD3) and B (CD79α) cell lines were present among them. Such forms of the leukocytes as monocytes (S100) and various forms of granulocytes (MPO) were also present in the immune microenvironment of the tumor. B lymphocytes were present in 20 to 60 % of the total number of leukocytes. We found that the number of T lymphocytes has been approximately 25–70 % of the total number of leukocytes. The total number of T and B lymphocytes was 85–90 %. The number of granulocytes was 1–7 %, macrophages were up 15 %. The antigen presenting monocytes were observed in most cases with B lymphocytes. An important aspect of the research is the study of the ratio between different types of leukocytes. Our study showed that in the case of steroid-negative breast cancer the ratio В-lymphocytes:T-lymphocytes:granulocytes:antigen presenting monocytes was an average as follows 6:2,5:0,5:1. It means that the number of B lymphocytes was more than 60 % and T lymphocytes were near 25 %. There was found the indirect correlation between the presence of B lymphocytes and expression of steroid receptors (*p* < 0.05). The ratio of imunocytes in steroid-positive breast cancer was an average as follows 2:7:0,5:0,5.

**Fig. 2:**
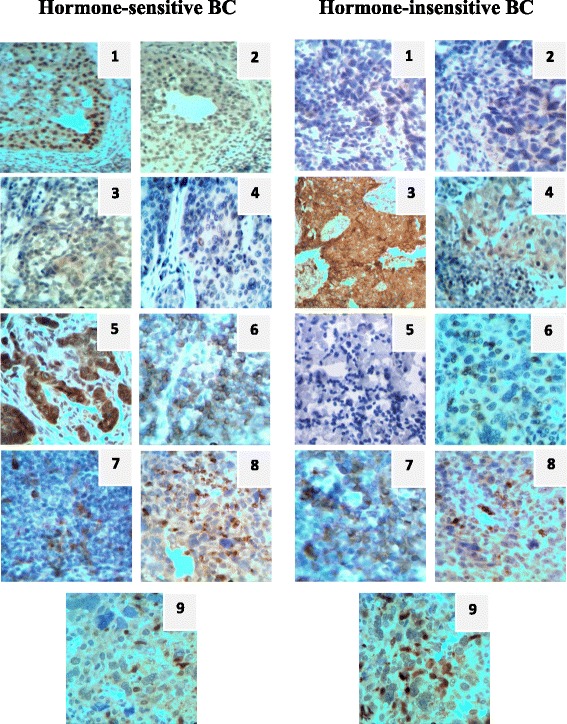
The immunohistochemical study of breast cancer tissue. 1 – ERα, 2 – PR, 3 - HER2/neu, 4 - Hsp90α, 5 - Bcl-2, 6 – CD3, 7 – CD79α, 8 – MPO, 9 - S100

Commending the above, we can state the fact that, depending on qualitative composition of the immune microenvironment the different expression of the prognostic markers are observed in breast cancer cells (Fig. 2).

Concerning PR, its quantity directly depends on the amount of ER. This indicates their simultaneous synthesis and dependence of PR from the presence and quantity of ER [4].

## Discussion

The progression of breast cancer depends on many factors, including the tumor microenvironment. The surrounding inflammatory infiltration in the list of prognostic factors is not the last place. We cannot exclude its effect on the expression of receptors. This is clearly seen in these types of breast cancer, such as medullary and inflammatory carcinoma, which are always accompanied by pronounced inflammatory infiltration in the microenvironment. They have the negative status of the receptors for steroid hormones in most cases.

In our opinion, there are several reasons for the appearance of immunocytes among the tumor cells. Therefore as a tumor is an immunologically foreign tissue, the macroorganism is constantly struggling with it. This is only possible on condition that different forms of leukocytes are involved in this process. On the other hand, the impact of exogenous factors that stimulate inflammation may also influence the course of carcinogenesis. For example an increased penetration of heavy metal salts into the body leads to their accumulation in tumor tissue. The activation of the immune system occurs by the stimulation of oxidase stress and increase of tissue calcification in which they take part.

Undoubtedly the inflammatory infiltration influences up the receptor expression, but as can be seen from our results it is not dependent on the type of infiltration. It depends on the qualitative composition of cellular microenvironment. The sequence of events is as follows. The macroorganism recognizing the tumor as a foreign substance starts primarily the cellular immunity. The more tumor cells are foreign the more this reaction is expressed. It consists of the activation of the antigen-specific cytotoxic T (CD3) lymphocytes by immunocompetent macrophages (S100), which can cause apoptosis of tumor cells. After that the apoptotic corpuscles are phagocytized by the segmented leukocytes (MPO). This process does not influence on the expression of ERα and PR.

We saw quite a different picture in the case of the involvement of the humoral immunity in the struggle against cancer. The antigen presenting cells recognize antigen presented by tumor cells and present it through IL-1 to Th2. Th2 through IL-3 activate B lymphocytes. The secretion of IL-1 is accompanied by the transition of NF-kB in the active form. NF-kB stimulates protein kinase B, also known as Akt, which inhibits the activity of forkhead box O3 (FOXO3A) [13]. NF-kB influences on ER expression through stimulation of EZH2 (enhancer of zeste homolog 2) activity [14] and the activation of BLIMP-1 (PR domain zinc finger protein 1) [15]. The reduction of the number of FOXO3A, increasing of the number of EZH2, Akt and BLIMP-1 inhibit ER transcription. As a consequence the expression of intranuclear receptors for estrogen is reduced. Based on this, we explain the absence of ER in our study when В lymphocytes predominate in the tumor microenvironment.

A correlation between the level of Hsp90α expression and the leukocyte infiltration in breast cancer stromal tissue shows the influence of the immune microenvironment on the presence of the heat shock proteins. His constant presence with HER2/neu positive breast cancer confirms his participation in tumor progression. Hsp90α stabilizes HER2/neu and modulates their effect [20]. It stimulates the resistance of breast cancer to HER2/neu directed drugs [21]. This may explain the fact why ERBB2 gene amplification is not always accompanied by the expression of HER2/neu, which may be linked to the lack of Hsp90α. This chaperone effects on ER and PR, on the one hand stabilizing their structure, on the other hand regulating activity of Akt and NF-kB [3, 22]. Hsp90 promotes the activation of anti-apoptotic protein kinases (bcl-2) and blocks APAF-1, the protein of internal apoptotic way, thereby bolstering the development of cell resistance to their programmed death [25]. Based on this, increasing the number of Hsp90 in the conditions of oxidase stress and inflammation leads to the increase of tumor cell resistance and assists in the expression of prognostically unfavorable receptors. Hsp90 worsens the prognosis of breast cancer because it also modulates the synthesis of such proteins as p53, angiogenic factor HIF-1a, VEGF and others, which are aggressive factors in the cancer process [26].

Previous studies have shown that Bcl-2 was expressed almost exclusively in ER+ diseases (about 85 %) because BCL2 is an estrogen-responsive gene [27]. The same results have been obtained in our studies in estrogen-positive breast cancer cases. It is believed that the presence of Bcl-2 receptors is a favorable prognostic factor [28]. While Bcl-2 has emerged as an important prognostic marker in breast cancer, its precise role as a predictive marker or therapeutic target is not known [29]. We believe that the presence of Bcl-2 protein is not an indicator of favorable course. This is one of the ways to improve the implementation of proliferative potential of cells through the ER. Inasmuch as a function of estrogen is the amplification of cell division, it can be implemented either through transcription of estrogen-dependent genes that are responsible for the separation or through activation of transcription estrogen-dependent genes that block apoptosis such as BCL-2. In our opinion the presence of Bcl-2 indicates a more aggressive course of breast cancer. Formerly it was considered as a prognostically favorable factor due to the presence in most cases the receptors of steroid hormones that are prognostically favorable markers of breast cancer.

Moreover the research has shown that therapeutic effect is enhanced in concurrent usage of the estrogen receptor inhibitors and BH3 mimetics, which have been developed to counteract antiapoptotic proteins such as Bcl-2 and Bcl-2-related proteins Bcl-XL and Bcl-W [30]. There is also NF-кB-dependent way of activation synthesis anti-apoptotic members of the Bcl-2 family (Bcl-2, Bcl-XL and BFL1) [17, 31]. And again we must remember about inflammation, IL-1 and their effects on NF-kB synthesis, through which they support resistance of cancer cells in the struggle for survival. That is why we observed isolated cases Bcl-2 positive breast cancer (weakly intense reaction) in cases of ER absence.

**Scheme 1:**
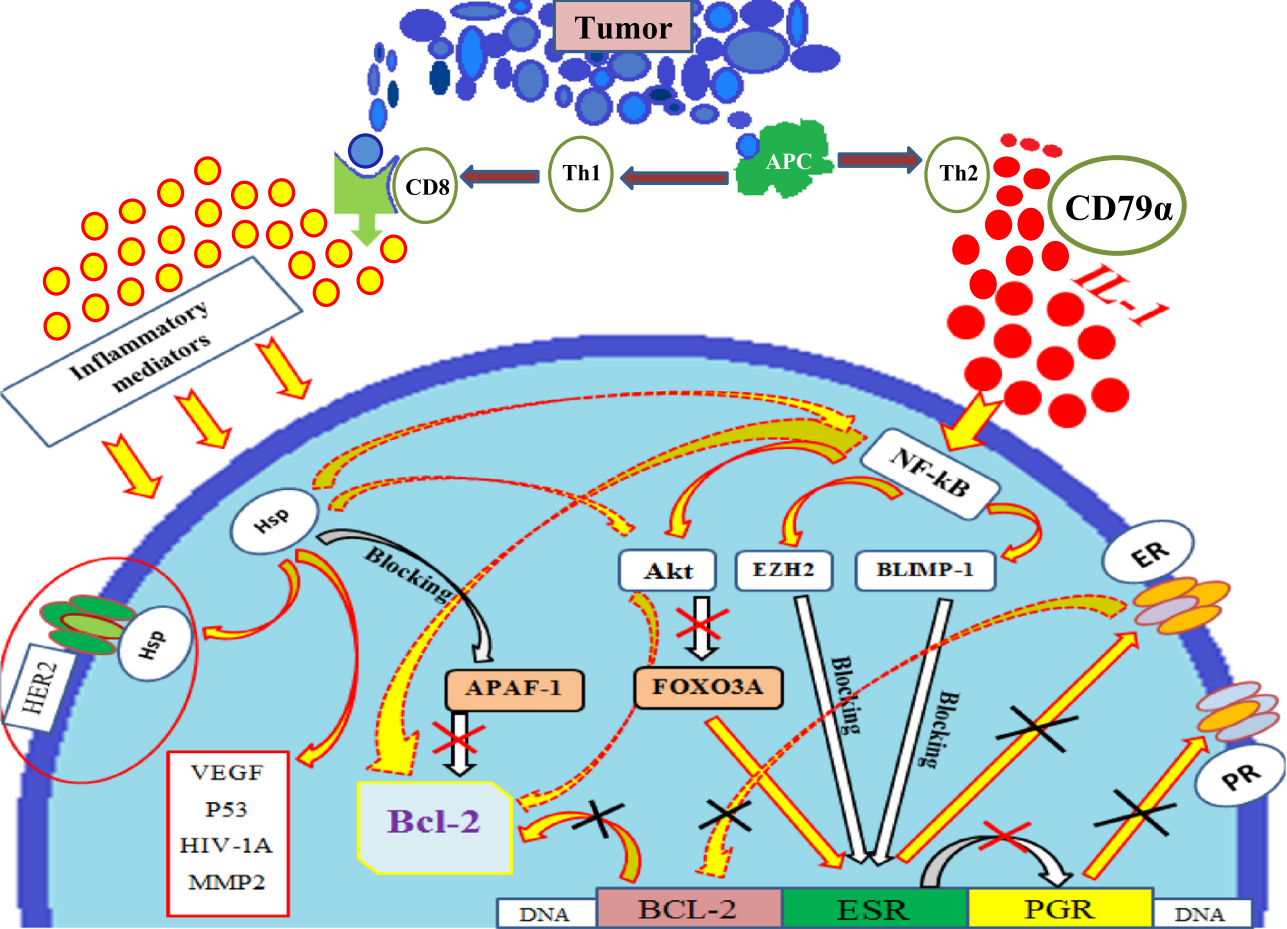
The influence of inflammation on the receptors of breast cancer cells. Tumor cells are recognized by the antigen presenting cells (APC) and the immune response is triggered. I. CD8 cells are activated by Th1. As a result of their interaction the inflammatory mediators are freed. They stimulate the accumulation of Hsp90 in a cell. Hsp90 stimulates several functions: 1) the stabilization of HER2/neu receptors; 2) the maturation and reparation of such proteins as VEGF, p53, HIV-1A, MMP2; 3) the blocking of APAF-1, which inhibit the function of Bcl-2; 4) the accumulation of Akt and NF-kB. II. APC secretes IL-1 that activates CD79 α (B lymphocytes) cells. IL-1 which is excreted by APC and CD79α cells induces the accumulation of NF-kB in cell. NF-kB starts the following processes: 1) the accumulation of Akt, EZH2 and BLIMP-1; 2) the activation of Bcl-2. Akt activates Bcl-2 and blocks FOXO3A, which is responsible for the transcription of ERS. EZH2 and BLIMP-1 also block the ERS transcription. As a result ER disappears. Because PR is formed after ER then they disappear too. BCL 2 is an estrogen-responsive gene, so the amount of Bcl-2 is also reduced

The general sequence of influence inflammation on the receptors of breast cancer cells is shown in Scheme 1.

Analyzing obtained data we can assert that immune microenvironment plays one of key roles in the progression of malignant process. This may explain those cases of breast cancer, when the cells with different phenotype of the receptor status can be found in one section. On the one hand, this means that the cells lose their ability to perceive signals from the macroorganism, on the other hand they adapt to new environment becoming more resistant. A similar situation occurs in metastases in the lymph nodes, where tumor cells are surrounded by B lymphocytes.

Immune system cells influence upon the course of cancer, but we haven’t found yet whether positive or negative. We consider that the immunocytes remove the influence of steroid hormones by blocking estrogen receptors as a factor of tumor initiation in the mammary gland. But in the future progress it begins to contribute to carcinogenesis progression.

## Conclusions

The immune microenvironment through the activation of intracellular mediators blocks ERS genes transcription. This circumstance is possible due to the presence of B lymphocytes in an inflammatory infiltrate. It leads to the disappearance of estrogen receptors, and as a result to the disappearance of progesterone receptors. The inflammation provokes the accumulation of Hsp90 in a cell. It contributes to the stabilization of HER2/neu receptors and most proteins that promote tumor progression. The lack of estrogen receptors is a factor of locking estrogen-dependent gene BCL-2 transcription and as a consequence to the decrease of Bcl-2. All of the above stimulates the breast cancer progression by providing tumor more malignant opportunities.
